# Supplementary material for: Targeted transcriptomic study of the implication of central metabolic pathways in mannosylerythritol lipids biosynthesis in Pseudozyma antarctica T-34
Source: PLoS One. 2020 Jan 10;15(1):e0227295. doi: 10.1371/journal.pone.0227295 (PMC6953796; doi:10.1371/journal.pone.0227295)
Supplement: Attachment — Submitted filename: Responses.docx [file pone.0227295.s005.docx]

**Reviewer #1**

>> Thank you for careful looks at our manuscripts. Followings are our answers to the comments and inquiries (the changes are highlighted).

Linguistic comments

**• Line 65: “large amounts of vegetable oil”. Change it to “vegetable oil”.**

>> As your comment, it was changed.

**• Line 153: “a large quantities of MELs.” Change it to “MELs” or define large quantities of MELs**

>> As your comment, it was changed to “MELs”.

**• Line 197: Remove “surprisingly”**

>> As your comment, it was removed.

**• Line 251: “were almost negative”. Change it to “close to zero”.**

>> As your comment, it was changed to “close to zero”.

**• Line 289: “enzymes”. Change it to “gene expressions”.**

>> As your comment, it was changed to “gene expressions”.

**• Line 314: “strongly induced”. Change it to “induced”.**

>> As your comment, “strongly” was omitted.

**• Line 338 & 352: “large quantities”; Change it to “larger quantities”.**

>> As your comment, they were changed to “larger quantities”.

**• General: When adding production numbers, they should be mentioned in volumetric productivities for improved comparison in literature.**

>> Thank you for your comment. We will revise the paper as much as possible so that we can respond to comments.

Research comments

• **There is no mentioning of any statistical significance of the genes or the selection procedure of the genes illustrated in figure 2 B&C. This part needs extended clarification.**

**• Line 177-181: What is the scientific background of this selection procedure for “similar genes expressions to the MEL biosynthesis genes”? Cut-offs of A-values > 14 and M < 0 for U. Maydis orthologs seem to be defined arbitrary. One of the MEL production genes has even a M>0. Revise the selection procedure or mention the scientific/statistical incentives for the cut-offs.**

>> Fig. 2B & C shows that *P. antarctica* MEL genes were expressed regardless of the carbon sources, and as a result, the A-values for the genes were high and the M-values were close to zero. On the other hand, in *U. maydis*, A-value of no gene exceeded 14 showing the expression level was low. The M-values of some genes were below -2, indicating that the MEL genes were regulated according to the carbon sources.

>> In the previous paper, another analysis, GSEA, suggested that the gene groups regulated by the carbon sources in the two fungi were different.

>> We wanted to know why MEL productions are high regardless of the carbon source in *P. antarctica*, so we decided to look for genes that behave similarly with MEL genes. For this reason, genes with an A-value of 14 or more were selected in *P. antarctica*. Furthermore, using the results of *U. maydis*, we tried to narrow down this gene group further. In *U. maydis*, no genes except one were expressed using oil as a carbon source, and the M-values were not high. Because *P. antarctica* and *U. maydis* share many orthologous genes, so it was possible to narrow down the *P. antarctica* genes using the results of *U. maydis* orthologous genes.

>> The gene group thus narrowed down might contain a lot of false genes, but an interesting result was obtained by looking at the list of the 264 genes.

**• There is no mention of exact replicate numbers for all the experiments performed.**

>> The explanation of previous microarray was described in previous paper [16].

>> Figure 5A: the explanation “A typical sample of three independent experiments was shown in each lane.” was added in the legend.

>> The explanation for measurements of wet cell weight was added in materials and methods as the independent paragraph.

>> The replicate number for enzyme activity is shown in the end of enzyme assay of materials and methods.

**• Line 310: Why was wet cell weight used and dry cell weight. Change in wet cell weight can be caused by changes in substrate concentrations or evaporation.**

>> As described above, the explanation was added in materials and methods.

Conclusion comments

**• Line 170: Can you compare A-values between two different organisms?**

>> The comparison was reported previous paper. In the revision, the reference number was added.

**• Line 211-212: State with literature or experimental evidence why TCA and glycolysis are related to production of MELs.**

>> Thank you for your comment. The sentence was revised to ”In addition, the genes related to glycolysis and the TCA cycle, which are part of the central metabolic pathway, may relate to MEL production, because the transcriptional regulatory characteristics are similar to MEL biosynthesis genes.”.

**• Line 265-267: It’s only a slight indication. There are more levels of regulation (post-transcriptional, translational, post-translational,…). This statement needs either metabolomic data or needs to be removed.**

>> According to your comment, this sentence was removed.

**• Line 326: change “resulting in” to “which could be the reason for”.**

>>As your comment, it was changed to “which could be the reason for”.

**• Discussion**

**Add comparisons with literature concerning the central metabolism of lipophilic fungi.**

>> As your comment, we added the description about lipophilic fungi utilizing a kind of volatile fatty acid, acetic acid.

**• Discussion: Add comparisons with literature concerning the carbon sources used for MEL production and their respective production numbers.**

>> As your comment, a paragraph regarding to carbon source and MEL production was added in Discussion.

Figure comments

**• Figure 1: Figure description doesn’t match the figure. Discrepant abbreviations and full names of enzymes.**

>> Thank you for your comment. It is description mistakes. Figure 1 was revised.

**• Figure 1: rarely does vegetable oil solely consist of C18 fatty acids. It should be made clear that the fed vegetable oil predominantly consists of C18 fatty acids.**

>> As your comment, it was revised.

**• Figure 2 B&C: Density is not clear. Use a continuous colour scale.**

>> Thank you for your comment. Present Figure 2 B&C have been depicted by continuous color scale based on the number of genes. However, the description was revised because the numbers were overlap.

**• Figure 2: “The genes responsible for MEL biosynthesis are overlaid on the hexplot”. Mention they are coloured in pink.**

>> As described above, the overlapped numbers were revised.

>> The explanations of dots in pink were added in the legend.

**• Figure 5 B: Can it be that the higher production is cause by higher biomass? Calculate specific productivities (g MEL/ (g Biomass*h).**

>> Sorry for the confusing expression. In Figure 5A, different amounts of samples were applied on TLC analysis. The vegetable oil sample is 2.5μL, on the other hand, glucose sample is 10μL. That means the productivity with vegetable oil is clearly higher than that with glucose. However, the Figure 5 was revised as well as some comments from other reviewer.

**• Figure 5 B & C: Define the error flags. Are they standard deviations, confidence intervals,…**

>> Description was added in the legend because of standard deviations.

Extra information/reference necessary

**• Line 58-60: “Pseudozyma antarctica T-34 produces large amounts of MELs when grown in culture containing vegetable oil as the carbon source, and the production yield reaches 140 g/L using n-alkanes as the carbon source”. Define “large amounts” and state with relevant research.**

**• Line 63: Define “lower amounts” and state with relevant research**

>> Thank you for your comment. This paragraph was revised, and explanation was added.

**• Line 75: Define “more effective”.**

>> Thank you for your comment. The sentence was revised to “In addition, MEL production from vegetable oil by *P. antarctica* T-34 (30 g/L of MELs, mainly MEL-A) was more effective than that of *U. maydis* UM521 (slight amounts of glycolipids on TLC analysis), suggesting that *P. antarctica* T-34 may utilize vegetable oil for growth and glycolipid synthesis. However, the characteristics of oil utilization by *P. antarctica* T-34 remained unclear [16].”

**• Line 124 & 127: “Tri-isocitrate”. Do you mean tri-sodium-isocitrate?**

>> Thank you for your comment, it’s description mistake. It was revised.

**• Line 137: URL is redundant information**

>> The URL was removed.

**• Line 144: Add the type of normalization that was used. In case of normalization with certain household genes, mention the genes used.**

>> As your comment, the brief explanation was added in “Materials and methods” section.

**• Line 158: “(more than 30 g/L)”. Mention the exact amount that was measured**

>> According to your comment, “more than 30 g/L” was revised to “30 g/L of MEL-A as the main component of MEL mixture”.

**• Line 159: amount of MELs produced by U. maydis should be define.**

>> According to your comment, the sentence was revised to “*U. maydis* UM521 produced only small quantities of MELs on TLC analysis, and considerable soybean oil remained in the culture [16]”.

**• Line 161: MEL production should be mentioned.**

>> As your comment, “1.0 g/L of mono-acylated MELs as the main components of MEL mixture” was added.

**• Line 167: “As described previously”. Add reference.**

>> The reference number was added.

**• Line 344: Quantify “large quantities” of MELs.**

>> The sentence was removed based on comment form other reviewer.

**Reviewer #2:**

**In the manuscript entitled „A novel aspect of the production of mannosylerythreitol lipids in Pseudozyma antacrtica T-34 based on gene expression of central metabolic pathways“ the authors Wada et al. have analysed micro array data of two fungi (P. antarctica T-34 and Ustilago maydis 521) and two growth conditions (glucose and soybean oil).**

**P. antarctica T-34 has produced under soy bean oil much more MELs than under glucose whereas in U. maydis the MEL production under soybean oil growth conditions is heavily repressed.**

**The authors have used the KOG nomenclature to categorize all genes and have identified genes of the primary metabolism to be upregulated in soybean oil conditions in P. antarctica. In contrast, most of these genes are down-regulated in U. maydis.**

**Finally, the authors demonstrate that under soybean oil conditions isocitrate dehydrogenase expression is increased and this coincides with increased activity.**

**The current work is interesting and will allow further manipulation of P. antarctica T-34 to increase MEL biosynthesis.**

**But there are some points necessary to improve the manuscript, the tables and the figures:**

>> Thank you for careful looks at our manuscripts. Followings are our answers to the comments and inquiries (the changes are highlighted).

**The title starts with “A novel aspect..” Please specify or change the title.**

>> The other reviewer also mentioned the same comment, and the title was changed to “Production characteristics of mannosylerythritol lipids in *Pseudozyma antarctica* T-34 based on gene expression of central metabolic pathways” according to your comment.

**Lane 20: industrial lipases, mannosyl…**

>> The sentence was revised to “…industrial lipases and mannosylerythritol lipids (MELs), which are multi-functional glycolipids.”.

**Lane 85: please complete the sentence: “…adapted to aerobically…”**

>> The sentence was revised to “…is adapted to aerobically produce…”.

**Lane 110: please specify the concentration of the NH4OH-solution used as solvent system**

>> Description of the concentration was revised to v/v. In our study, 2ml of NH4OH-solution was used in 100 ml of the solvent system.

**Lane 150: The title “Pseudozyma antarctica genes are expressed…” is confusing. What kind of genes do you mean? Not all genes are expressed with high intensity, are they?**

>> According to your comment, the title was revised to “Pseudozyma antarctica genes expressing under oily conditions.

**Table 1: I guess that you have ordered the table according to the Alphabet of the description. I would prefer to have then the description in the first column (not in the second). For the metabolism section “Energy production…” should be reordered. The row “Total CDS” should be changed with the row “The genes categorized…”. You have mentioned in the entire table only the KOG classified genes (93+42+16+28=179)**

>> As your comment, these were re-sorted.

**Lane 241: remove “Consequently,”**

>> As your comment, it was removed.

**Lane 258 spelling: Antarctica**

>> It was revised.

**Lane 297: “large amounts of MELs”: can you specify, which variants of MELs are produced?**

>> The main product is MEL-A, so the words “mainly MEL-A” was added. Also, corresponding figure was revised according to the other reviewer comment. In addition, the legend was revised, because the description about used sample volume was description mistake.

**Lane 311: …isocitrate dehydrogenase (NAD+)…” In the figure you use MaxV. Please clearify.**

>> Sorry, it is a description mistake, so I corrected the description in the figure.

**Lane 317: “…no activity was noted under oily conditions.**

>> The sentence was revised to “the activity was low in the presence of glucose, and no activity was noted under oily conditions.”.

**Figure 1: According to the legend (Lanes 54-56) the proteins are assigned differently (Emt1p not EMT1 and so on). The proteins involved in the step from mono-acylation to di-acylation are the acyl-transferases MAC1 and MAC2 (Mac1p/Mac2p). In your scheme acylation of C3 (by Mac2p) precedes the acylation of C2 (by Mac1p). Please comment on that in the introduction.**

>> Thank you for your correction. These are description mistakes. Figure 1 was revised.

**Figure 3: “vegetable oil over glucose”**

>> The description was revised from “vegetable oil for glucose” to “vegetable oil over glucose”.

**Figure 4: Great work!!**

>> Thank you for your encouragement.

**Figure 5:**

**A: Please add MEL-A, -B, -C; the curly bracket for MELs is huge. Are you sure, that the slow migrating bands are MELs?**

>> As your comment, the figure was revised.

>> All of the blue bands are isoform of MELs based on our previous studies.

**Why you mention “Olive oil” and not “Soybean oil”?**

**B: again “Olive oil”**

>> Thank you for your comment. Figure 5 was revised because of description mistake.

**C: again “Olive oil”. I propose to use different colors as in 5B, because you performed a different experiment.**

>> As your comment, the colors of C were changed as well as B.

**Lane 339-344: I think you can remove the sentence: “Hence, ….quantities of MELs”. To my opinion it repeats the sentence before.**

>> As your suggestion, the sentence was removed.

**Lane 383: “..will help to identify…”**

>> It was revised.

**Supplemental tables:**

**As for table 1: The sorting criteria must be in the first column.**

>> As your comment, these were re-sorted.

**S1: Start with KOG, KOG number, Pa gene…**

>> As your comment, these were re-sorted.

**S2 and S3: please use either upper- or lowercase names for the enzyme description and resort according to Alphabet, then EC-number…**

>> As your comment, these were re-sorted.

**Legend: …*3 BBH means bi-directional best hit. Plural means the homology…” Please change all “pulural” to plural in column Type of S2**

>> As your comment, these were changed.

**S4: please sort the gene numbers: 1, 2, 3, 4, 5, 6, 7, 8, 9, 10, 11, and so on
Start with: 1c00036, 1d00040, 2c00011 and so on**

>> As your comment, these were re-sorted.

**The description column uses cryptic abbreviations e.g. row 9: Aldedh ? Please use for all domain abbreviations the full form.**

>> As your comment, these were renamed.

**Reviewer #3:**

**The manuscript "A novel aspect of the production of mannosylerythritol lipids in Pseudozyma antarctica T-34 based on gene expression of central metabolic pathways" gives new insights on the genetic and metabolic regulations controlling mannosylerythritol lipids (MEL) biosynthesis in two yeasts. The manuscript is providing a new piece o knowledge ans is sound for publications, yet after considering the following comments:**

>> Thank you for careful looks at our manuscripts. Followings are our answers to the comments and inquiries (the changes are highlighted).

1- **Lines 344-345: The authors mention that the Ustilago maydis had poor growth when using vegetable oils as carbon source, which is not the case with Pseudozyma antarctica. I strongly suggest the absence of lipases and esterases in U. maydis which explain their poor growth and low MEL production. Therefore, I suggest the authors screen the genome of U. maydis for lipases and esterases and if absent, to use this as the explanation of the poor growth and low MEL production. Also, does U. maydis grow on alkanes or fatty acids or glycerol separately?**

>> Thank you for your comment. In our previous genome analysis, the both of strains conserve several predicted genes for lipases, and the details of that matters are still unclear. Therefore, we think that the relationships between lipase and oil assimilation in U. maydis aren’t describe in this paper, that is, further study is require to discuss about that.

>> The details of utilization of alkanes, fatty acids and glycerol are unclear, but in my knowledge, glycerol is an available carbon source for growth of *U. maydis*. At least, in our study, *U. maydis* doesn’t grow in a medium containing vegetable oil as a carbon source. As your suggestion, cultivation of *U. maydis* with fatty acids or alkane may reveal if *U. maydis* fails to degrade vegetable oil. It is further interesting investigation for us.

>> On the other hand, we previously reported poor growth and MEL production with vegetable oils in U. maydis. Therefore, in the revision, “…identical to our previous report [16].” was added in Discussion.

2- **Line 67-68: Although authors are referring to reference 15, yet it would be easier for the reader to know briefly how the genomic and transcriptomic analyses led to the conclusion that P. antarctica is oleaginous.**

>> Thank you for your suggestion. As your comment, the brief explanation was added in Introduction.

3- **Lines 198-199: hard to read. it is better to say "metabolism related genes" rather than "metabolism genes"**

>> As your comment, it was revised to “metabolism related genes”.

4- **Line 253: glyocylate to glyoxylate.**

>> It was revised.

5- **it is strongly recommended to show the expression fold changes of MEL biosynthetic genes and transport in a single separate figure similar to figure 3.**

>> Thank you for your suggestion. The explanation using the M-value averages of MEL biosynthesis genes was added in the result section, because the M-value is too high compared to the other genes in Figure3.

6- **English style and grammer needs some improvement for a more easy reading.**

>> Thank you for your comment. Although English review of the manuscript was done, we received some comments from other reviewers about that, and revised according to each comment.

**Reviewer #4:**

**The results embody this manuscript is a continuation of their previous studies, and has merit. This manuscript can be published with some minor modifications. The suggested changes are as follows:**

>> Thank you for careful looks at our manuscripts. Followings are our answers to the comments and inquiries (the changes are highlighted).

**Full Title: Production characteristics of mannosyl.....**

>> The title was changed to “Production characteristics of mannosylerythritol lipids in *Pseudozyma antarctica* T-34 based on gene expression of central metabolic pathways”.

**Short Title: Metabolic transcriptomics.....**

>> The short title was changed to “Transcriptomic analysis relating glycolipid production”.

**Abstract L34-36: These results suggest that central metabolism of.......**

>> It was revised.

**L85-86: These results ... ....... ...... modification of its central metabolic system.**

>> It was revised.

**L386-391: Based on the transcriptomic characterization and biochemical changes - the authors concluding statements are far and wide or out of focus.**

>> As your suggestion, the last sentence was revised to “Further genetic study will facilitate not only the development of useful industrial strains as MEL producer by genetic modification, but also improve our understanding of phytopathological mechanisms and environmental adaptation of these basidiomycetous genera.”.
