# Supplementary material for: Targeted transcriptomic study of the implication of central metabolic pathways in mannosylerythritol lipids biosynthesis in Pseudozyma antarctica T-34
Source: PLoS One. 2020 Jan 10;15(1):e0227295. doi: 10.1371/journal.pone.0227295 (PMC6953796; doi:10.1371/journal.pone.0227295)
Supplement: Attachment — Submitted filename: response_R.docx [file pone.0227295.s006.docx]

**Reviewer #1**

**Most comments were resolved still some aren’t.**

>> Thank you for your review. Explanations and corrections to your comments are as follows.

1) **There are claims about higher productivities and higher growth but these are not supported with the right data. MEL production is measured by TLC which is a qualitative measurement technique and not a quantitative one. I have the feeling that the author cannot or won't quantify there MEL's with an adequate technique (e.g. HPLC-ELSD). Biomass growth is measured with cell wet weight after MeOH extraction. There are so many biasses on this technique (substrate, lipid extraction by MeOH, evaporation, cell size, lipid content,....). So I suggest, either you analyze your production parameters (in g/(L*h)). If this is no option than you will have to remove your claims of higher production or state that a TLC analysis based on visual comparison can only give a small hint for higher concentration. Biomass should be measured with another technique than cell wet weight like e.g. cell dry weight after removal of product by ethyl acetate washing. (don't use MeOH, ethyl acetate will evaporate). If there is a difference in biomass than should the productivity be linked to the biomass as well (specific productivity). This will probably result in interesting findings.**

>> Thank you very much for your kind suggestion. As your comment, we didn’t quantify the amounts of MELs by HPLC in this study, because it is known that *Pseudozyma antarctica* is able to produce larger amounts of MELs from vegetable oils than glucose. However, TLC analysis doesn’t give quantitative result. Therefore we added the following explanation in this revision (P6L119-123). “Although not a quantitative method, the MEL production was evaluated by TLC analysis in order to make a simple visual comparison of differences in MEL production depend on the producers and substrates.”

>> As your comment, actually dry cell weight is better than wet cell weight to show the biomass, while similar tendency between wet cells and dry cells have been obtained in our previous research experience. Also, further interesting finding may be obtained in more precise analysis as your suggestion. On the other hand, the aim in this study is to show the relationship between the difference in substrates and producers on the growth of produces. Therefore, “To estimate wet cell weight” was revised to “To estimate the effect of cell growth on different substrate” (P6L129). Also, the title “Wet cell weight” was revised to “Estimation of cell growth” (P6L128).

2) **Figure 2 B&C: As described in your article, “Genome and transcriptome analysis of the basidiomycetous yeast Pseudozyma antarctica producing extracellular glycolipids, mannosylerythritol lipids”, there were gene transcriptions who were significantly different (p-value <0.005). Can you state that you only used these ones for further analysis when you were using the selection criterium M<0. This will improve your research by confirming that you lowered the false positive rate.**

>> We have included probability values (p-values) for the picked 264 genes in Table S1. We found 140 genes (54%) of the 264 genes had p-values lower than 0.05 for the U. maydis ortholog and check the 140 genes.

>> We found that the ratio of the genes categorized in Metabolism was 45.7% and higher than the corresponding ratio written in the Table 1 (35.2%). On the other hand, lots of genes categorized in “Cellular processes” and “Information storage and processing” had p-values higher than 0.05, and the ratios of the genes for the two categories were less than the corresponding ratio written in the Table 1; 13.6% for the “Cellular processes” was less than 15.9% and 2.9% for “Information storage and processing” was less than 6.1%. Now we have included the p-values in the Table S1, the readers can check the number and ratio of genes in each category using the p-values in the Table S1. We appreciate your comment.

3) **Line 372 & 387: change large amounts to larger amounts or just to “presence of vegetable oil”.**

>> As your comments including 1), we revised them as follows.

(P5L95, P8L180, P17L322, P17L325)

“large amounts” were revised to “larger amounts”.

(P15L279, P19L377-378)

“large amounts” were omitted.

4) **381: precious report should be changed to previous**

>> As your comment, it was changed.

5) **Figure 5 A: the µl of sample should be under the respective lane for clarity.**

>> As your comment, the position of µl of sample was revised.

**Reviewer #2**

**Dears Authors, most of my concerns have been addressed in your revised version. But there are still two points which have not been answered satisfactory.**

>> Thank you for your comment. We revised the two points according to your comments.

**Figure 1: There is still a mistake in the biosynthesis pathway. Please look at Hewald et al. (2006) or Deinzer et al. (2019). Mat1 acetylates C4 and C6 residues of the mannose and not as depicted C2. I propose to cite these articles as basis for your figure. Do you have any hints that Mac2 acylates first then Mac1, or it is still done in a cooperative manner? Please change the figure accordingly to the literature.**

>> Sorry for the incomplete correction. We submitted the Figure before correction by mistake. As your mention, mat1 acetylates C4 and C6 residues of the mannose, and the order of acylation by mac1 and mac2 is unclear. We revised the Figure 1 in this revision.

**The concentration (molarity) of NH4OH-solution is still missing.**

>> Sorry for not understanding your previous comment. The description was revised to “chloroform-methanol-7 N ammonium hydroxide (65:15:2, vol/vol/vol).

**Reviewer #3**

**The manuscript looks much better and is sound for publication in its current status. Yet I suggest the title be changed from “Production characteristics of mannosylerythritol lipids in Pseudozyma antarctica T-34 based on gene expression of central metabolic pathways” to “Targeted transcriptomic study of the implication of central metabolic pathways in mannosylerythritol lipids biosynthesis in Pseudozyma antarctica T-34”**

>> Thank you for your suggestion. We revised the title because the proposed one is well.
